# Molecular mechanisms and differences in lynch syndrome developing into colorectal cancer and endometrial cancer based on gene expression, methylation, and mutation analysis

**DOI:** 10.1007/s10552-021-01543-w

**Published:** 2022-02-11

**Authors:** Hongfeng Li, Liwei Sun, Yan Zhuang, Caijuan Tian, Fang Yan, Zhenzhen Zhang, Yuanjing Hu, Pengfei Liu

**Affiliations:** 1 Department of Clinical Laboratory, Tianjin Academy of Traditional Chinese Medicine Affiliated Hospital, Tianjin, 300120 China; 2 Department of Interventional Oncology, Tianjin Huanhu Hospital, Tianjin, 300350 China; 3 Key Laboratory of Cancer Prevention and Therapy of Tianjin, Tianjin’s Clinical Research Center for Cancer, Department of Colorectal Oncology, National Clinical Research Center for Cancer, Tianjin Medical University Cancer Institute and Hospital, Tianjin, 300060 China; 4 Tianjin Marvel Medical Laboratory, Tianjin Marvelbio Technology Co., Ltd, Tianjin, 300381 China; 5 Department of Gynecological Oncology, Tianjin Central Hospital of Gynecology & Obstetrics, No. 156 Nankaisan Road, Nankai District, Tianjin, 300100 China; 6 Department of Oncology, Tianjin Academy of Traditional Chinese Medicine Affiliated Hospital, No. 354 Beima Road, Hongqiao District, Tianjin, 300120 China

**Keywords:** Lynch syndrome (LS), Colorectal cancer (CRC), Endometrial cancer (EC), Development mechanism

## Abstract

**Purpose:**

The aim of this study was to screen biomarkers specific to Lynch syndrome (LS) with colorectal cancer (CRC) or endometrial cancer (EC) to explore the mechanisms by which LS develops into CRC and EC and their differences.

**Methods:**

Differentially expressed or differentially methylated genes and differential mutations were identified in 10 LS, 50 CRC, and 50 EC patients from TCGA, and genes overlapping between LS and CRC or EC (named SGs-LCs and SGs-LEs, respectively) were identified. Afterward, we annotated the enriched GO terms and pathways and constructed a protein–protein interaction (PPI) network. Finally, samples from 10 clinical cases with MSI-H/MSS CRC and EC were collected to verify the mutations and their correlations with five LS pathogenic genes in the SGs-LCs and SGs-LEs.

**Results:**

A total of 494 SGs-LCs and 104 SGs-LEs were identified and enriched in 106 and 14 GO terms, respectively. There were great differences in the gene count and enriched terms between SGs-LCs and SGs-LEs. In the PPI network, *SST*, *GCG*, *SNAP25*, and *NPY* had the highest degree of connection among the SGs-LCs, and *KIF20A* and *NUF2* had the highest degree of connection among the SGs-LE. In the SGs-LCs and SGs-LEs, the genes whose expression levels affected the survival of LS, CRC or EC patients were quite different.

**Conclusions:**

*COL11A1* was found to be mutated in MSS CRC patients, similar to the mutations of *MSH6*. *SST*, *GCG*, *SNAP25*, and *NPY* may be biomarkers for the development of LS into CRC, and *KIF20A* and *NUF2* may be markers of LS developing into EC.

**Supplementary Information:**

The online version contains supplementary material available at 10.1007/s10552-021-01543-w.

## Introduction

Lynch syndrome (LS), also known as hereditary nonpolyposis colorectal cancer (HNPCC), is an autosomal dominant genetic disease caused by defects in DNA mismatch repair (MMR) genes. LS is the most common hereditary colorectal cancer syndrome, with clinical features of early onset and tumor susceptibility in the proximal colon [1, 2]. According to different clinical manifestations, LS can be divided into two categories: LS I and LS II. The only malignant tumor produced by LS I is colorectal cancer (CRC). In LS II, however, in addition to CRC, HNPCC-related parenteral tumors can also occur, such as adenocarcinoma in the stomach, endometrium, pancreas, or bile duct and malignant tumors of the blood system, among others [3]. Approximately, 5–6% of cases of CRC are associated with germline mutations [4]. Patients with hereditary CRC syndromes, such as LS and familial adenomatous polyposis, have a significantly elevated risk of CRC compared with the general population.

LS is the most common cause of hereditary CRC and the only known cause of hereditary endometrial cancer (EC). Approximately, 2% of EC cases are related to LS. EC is the first manifestation of disease in more than 50% of women with LS. These patients’ 10-year risk of developing EC is 26% [5], and the lifetime risk is 15–60% [6], which is greater than that of CRC [7]. With the increased popularity and level of detail of genetic testing, the pathogenic genes of LS have been clarified. The main reported genes are *MLH1*, *MSH2*, *MSH6*, *PMS2* (MMR genes), and *EPCAM* (non-MMR gene) [8]. Germline mutations in MMR genes cause microsatellite instability (MSI) and result in the loss of the corresponding MMR protein, thus affecting DNA mismatch repair function and increasing the risk of malignant transformation.

Although the pathogenesis of LS has been gradually elucidated, the molecular mechanism of LS developing into CRC or EC has been less reported, and l few articles have only mentioned that Lynch syndrome patients will develop CRCs as well as some endometrial tumors for clinical spectrum overlap [9]. Therefore, this study sought to identify genes specific to LS with CRC or EC by differential expression analysis, differential methylation analysis, gene mutation detection, and correlation analysis with the pathogenic genes of LS. Subsequently, through functional enrichment analysis, survival analysis, and clinical sequencing data verification, we clarified the underlying mechanisms of these genes in the development of LS into CRC or EC and their differences. Our study may provide new insights for the early screening and prevention of CRC and EC related to LS.

## Materials and methods

### Data collection and processing

The transcriptome sequencing data, methylation profiles, tissue mutation data, and survival data of 10 LS, 50 CRC, and 50 endometrial carcinoma patients were downloaded from The Cancer Genome Atlas (TCGA, https://tcga-data.nci.nih.gov/docs/publications/tcga/) as the training group. The sequencing platform used for the methylation profile was an Illumina HumanMethylation 450 BeadChip.

The transcriptome sequencing data were normalized using the normalize.quantiles function in the preprocessCore V1.32.0 package (http://www.bioconductor.org/packages/3.2/bioc/html/preprocessCore.html); then, log2 transformation was performed, and negative values were taken as 0. The org.Hs.eg.db package was used to map the Ensembl ID to the gene symbol, and probes without corresponding genes were removed. For duplicate gene IDs, the average expression value was taken as the gene expression value.

#### Differential expression, differential methylation, and mutation analysis

The differentially expressed genes (DEGs) in LS, CRC, and EC were identified with limma V3.32.2 (http://www.bioconductor.org/packages/3.5/bioc/html/limma.html) and recorded as DEGs-L, DEGs-C, and DEGs-E, respectively. The threshold criteria were |log (fold-change)|> 1 and *p* < 0.05.

The methylation profiles of LS, CRC, and EC were normalized using the preprocessCore V1.32.0 package. Subsequently, a *t* test was performed on the *β* value of each probe to find probes with *p* < 0.01 and |Δ*β*|> 0.05. The probes were mapped to the gene symbol using the IlluminaHumanMethylation450kanno.ilmn12.hg19 package (http://www.bioconductor.org/packages/release/data/annotation/html/IlluminaHumanMethylation450kanno.ilmn12.hg19.html), and duplicate gene names were removed. Finally, the differentially methylated genes (DMEs) were obtained and recorded as DMGs-L, DMGs-C, and DMGs-E.

The percentage of each mutation type in each group of patients was counted, and the genes with mutation frequencies greater than the median were further screened as high-frequency mutation genes. Genes with two or more mutations in one patient were counted once.

#### Screening of genes highly related to LS pathogenic genes in DEGs and DMGs

The DEGs and DMGs highly related to the pathogenic genes of LS (*MLH1*, *MSH2*, *MSH6*, *PMS2*, and *EPCAM*) were screened. For the highly related DEGs of LS and CRC, the correlation coefficient between each pathogenic gene and each DEG-L/DEG-C was calculated. Subsequently, the genes with | *r* |> median(|*r*|), where *r* represents the correlation coefficient, were taken as the highly correlated genes. The highly correlated genes in DEGs-L and DEGs-C were intersected to obtain the genes highly correlated with the pathogenic gene in both Lynch syndrome patients and CRC patients. Then, we obtained five sets of DEGs with high correlation with the five pathogenic genes. These DEGs were combined as DEGs-LC, which were highly related to the five pathogenic genes and specific for LS and CRC. Similarly, we acquired DEGs-LE (DEGs from DEGs-L and DEGs-E and specific for LS and EC), DMGs-LC, and DMGs-LE. Finally, the DEGs, DMGs, and mutations specific to LS and CRC (or EC) were obtained and named SGs-LC (or SGs-LE).

### Functional enrichment analysis

Functional and pathway enrichment analyses of SGs-LC and SGs-LE were performed via the Database for Annotation, Visualization, and Integrated Discovery (DAVID) V6.7 (http://david.abcc.ncifcrf.gov/) and Kyoto Encyclopedia of Genes and Genomes (KEGG) pathway (http://www.genome.jp/kegg). Gene Ontology (GO) terms and pathways with *p* < 0.05 were selected.

### Construction of protein–protein interactive network

The interacting protein–protein pairs within SGs-LC and SGs-LE were identified via STRING V10.5 (https://string-db.org/). Ultimately, the regulated network was established based on the protein–protein pairs and visualized by Cytoscape V3.5.1 software (http://www.cytoscape.org/download.php). The degree and betweenness of each node were counted by using CentiScaPe (http://apps.cytoscape.org/apps/centiscape), and the node with the largest degree of connectedness was found as the hub node.

### Survival analysis

The survival package in R 3.4.4 was used to calculate the significance of the effect on survival. For the genes with high node degrees in the network, genes were considered highly expressed in the patient when the expression value was greater than the median expression value; otherwise, it was low. Accordingly, the patients were divided into group-high and group-low. The survdiff command [10] was then used to analyze significant differences in the overall survival rate between the two groups. A *p* value < 0.05 was considered statistically significant.

### Validation of the specific DEGs

MSI was detected in CRC and EC patients. A total of 10 cases of CRC and 10 cases of EC with high-level microsatellite instability (MSI-H) were selected from Tianjin Central Hospital of Gynecology and Obstetrics, Tianjin Huanhu Hospital, and Tianjin Medical University Cancer Institute and Hospital; 10 cases of CRC and 10 cases of EC with microsatellite stability (MSS) were also selected. These patients served as the validation group and were then subjected to next-generation sequencing to detect gene mutations. Subsequently, mutations in SGs-LCs and SGs-LEs with high node degrees were verified in these four groups of patients, and their correlation with dislocation mutations in *MLH1*, *MSH2*, *MSH6*, *PMS2*, and *EPCAM* was also verified.

Afterward, gene set enrichment analysis (GSEA) (https://www.gsea-msigdb.org/gsea/index.jsp) was used to verify the scores of SGs-LC and SGs-LE with high node degree in gene sets related to LS and CRC (LS and EC). Nom *p* < 0.05 and FDR *q* < 25% were the criteria considered to indicate significant differences.

### Statistical analysis

SPSS Statistics version 21 (SPSS Inc., Chicago, IL, USA) was used for data analysis. Continuous variables were compared between groups by *t* test, while categorical variables were expressed as number and frequency and compared by chi-square test or Fisher’s exact test.

## Results

### DEGs, DMGs, and gene mutations

**Fig. 1:**
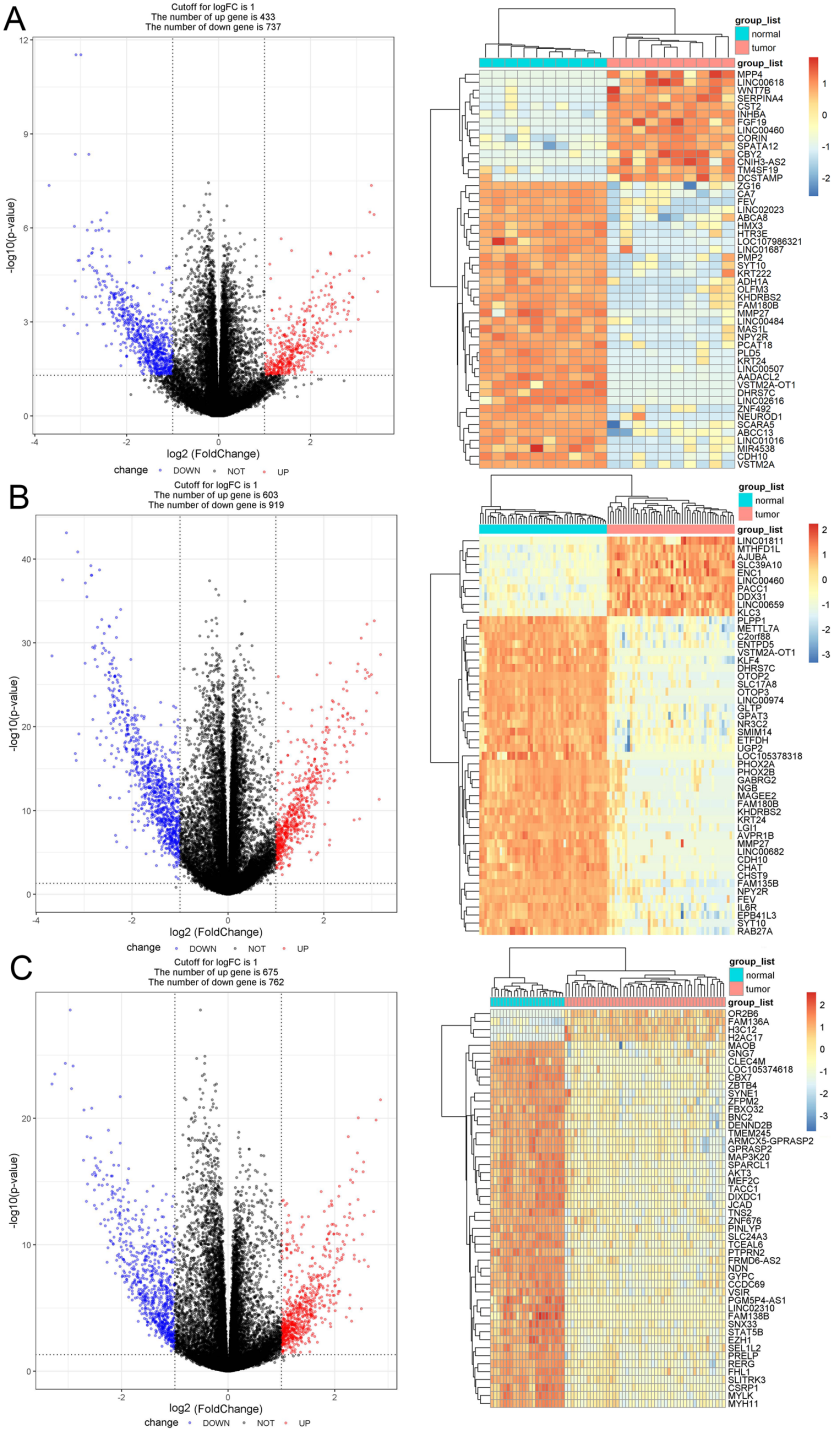
Cluster analysis of DEGs in Lynch syndrome, colorectal cancer, and endometrial cancer. The volcano plot and heatmap of DEGs-L (**a**), DEGs-C (**b**), and DEGs-E (**c**) showed that the expression of the 3 sets of DEGs was significantly different between tumor and normal tissues. Blue, red, and black spots represent downregulated, upregulated, and nonsignificantly expressed genes, respectively. DEGs, differentially expressed genes; DEGs-L, DEGs in Lynch syndrome; DEGs-c, DEGs in colorectal cancer; DEGs-E, DEGs in endometrial cancer

**Fig. 2:**
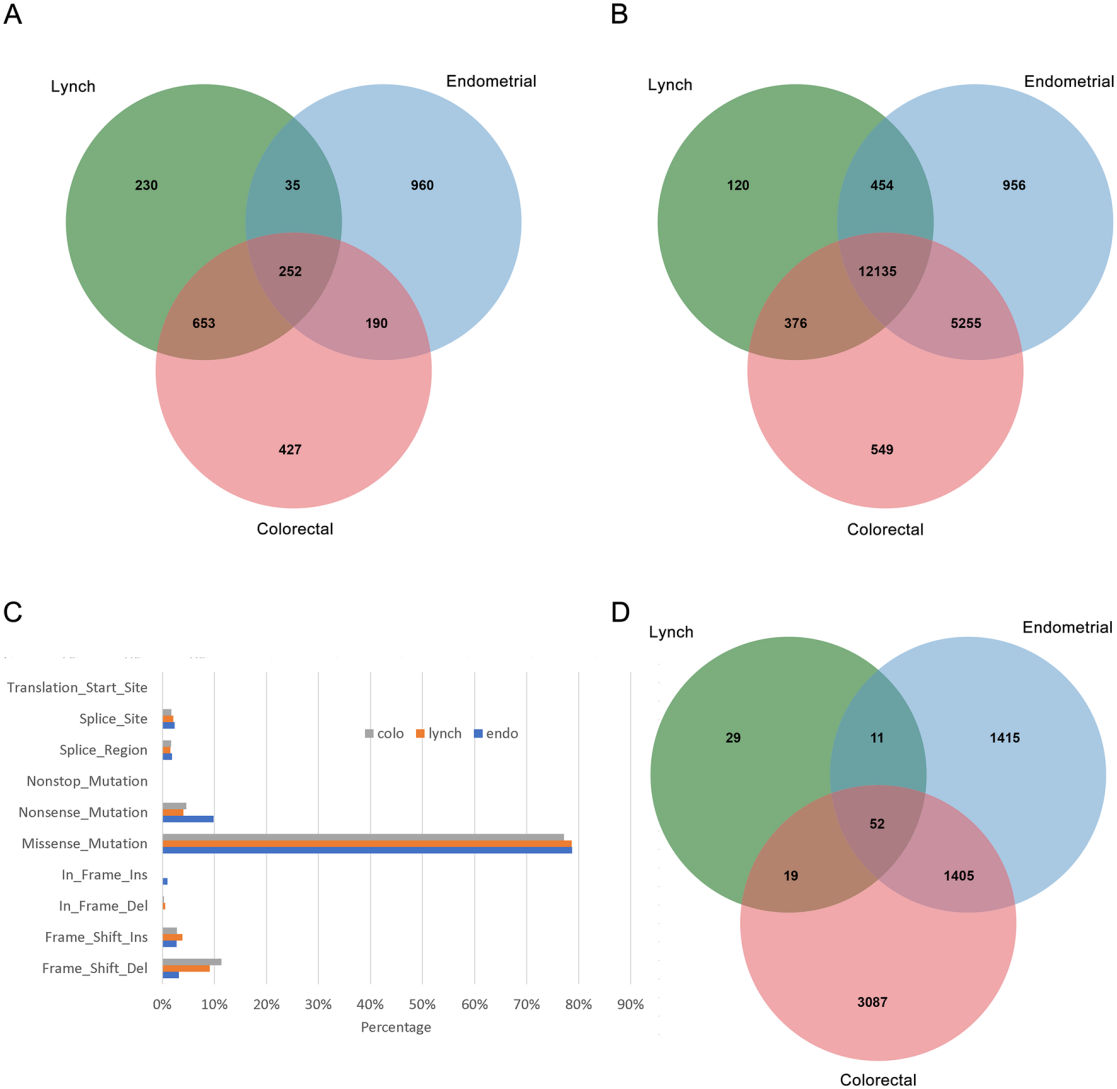
Venn diagrams of DEGs (**a**), DMGs (**b**), mutations (**d**), and the percentages of mutation types (**c**) in Lynch syndrome, colorectal cancer, and endometrial cancer. DEGs, differentially expressed genes; DMGs, differentially methylated genes

A total of 1170 (433 upregulated and 737 downregulated), 1522 (603 upregulated and 919 downregulated), and 1437 (675 upregulated and 762 downregulated) DEGs were obtained in LS, CRC, and endometrial carcinoma patients, respectively, and called DEGs-L, DEGs-C, and DEGs-E. Figure 1a–c represents the gene expression in total and the heatmap of the top 50 DEGs according to the *p* value through cluster analysis. Cluster analysis involves grouping a set of characteristics in such a way that objects in the same group (a cluster) are more similar (in some way) to each other than to the objects in other groups. We found that there was a substantial difference in the 3 sets of DEGs between tumor tissue and normal tissue. Among the above 3 sets of DEGs, there were 653 specific genes for LS and CRC, 35 for LS and EC, and 252 for LS, CRC, and EC (Fig. 2a).

By analyzing the methylation data, we acquired differentially methylated genes (DMGs). A total of 13,085 DMGs in LS (DMGs-L), 18,315 in CRC (DMGs-C), and 18,801 in EC (DMGs-E) were obtained. There were 376 DMGs in both LS and CRC, 454 common DMGs in LS and EC, and 12,136 overlapping DMGs among the three diseases (Fig. 2b). As shown in Fig. 2a, b, the number of specific DEGs in LS-CRC was significantly higher than that in LS-EC, while the number of specific DMGs in LS-EC was slightly higher than that in LS-CRC, indicating that the pathogenic processes connecting LS to CRC and EC might be different.

In the analysis of the mutation data, we counted the number and types of mutations in three patients (Fig. 2c, d). Among the three diseases, missense mutations were the most common, followed by frameshift-del and nonsense mutations (Fig. 2c). The differences in mutation type among the three diseases included that CRC had a higher proportion of frameshift-del mutations than EC and that the percentage of nonsense mutations was higher in EC than in CRC, illustrating that CRC and EC may occur and develop through different types of mutations.

We further counted the common mutations whose mutation frequency was greater than the median (median mutation of LS and CRC was 1 and EC mutation was 2). There were 111 high-frequency mutations in LS, 4563 in CRC and 2883 in EC. The common mutated genes in LS and CRC were screened, and the genes mutated in EC were excluded. Thus, specific high-frequency mutated genes in LS and CRC (Mut-LC) were obtained, with a total of 19 genes. Similarly, 11 common mutated genes were obtained in LS and EC (Mut-LE) (Fig. 2d). The number of specific mutations in both groups was small.

### Identification of DEGs and DMGs highly related to LS pathogenic genes

A total of 460 DEGs in both DEGs-L and DEGs-C (DEGs-LC) were obtained by analyzing the correlation between DEGs and the 5 LS pathogenic genes, as well as 24 DEGs in DEGs-L and DEGs-E (DEGs-LE). In the three groups of DMGs, we obtained 15 specific DMGs (DMGs-LC) of LS and CRC and 64 specific DMGs (DMGs-LE) of LS and EC, which were highly correlated with 5 LS pathogenic genes.

Then, DEGs-LC, DMGs-LC, and Mut-LC (DEGs-LE, DMGs-LE, and Mut-LE) were combined, and duplicate genes were deleted. Ultimately, 494 specific genes for LS and CRC (SGs-LC) were obtained, including *PTCHD1*, *SYT4*, and *COPDA1*, and 99 LS and endometrial-specific genes (SGs-LE) were obtained, including *CDC20B*, *SLC10A4*, and *LY6K* (Supplementary Table 1). The top 20 SGs-LC and SGs-LE according to *p* value are listed in Table 1. The numbers of SGs-LC and SGs-LE were quite different. Thus, it is speculated that LS might develop into CRC in more complex ways.

**Table 1:** The top 20 SGs-LC and SGs-LE according to *p* value

Group	Gene	logFC	*p* value	Gene	logFC	*p* value
SGs-LC	AADACL2	− 3.104	1.88E − 16	CDH10	− 2.608	1.60E − 09
	LINC00507	− 2.998	2.32E − 16	PCAT18	− 2.674	1.70E − 09
	DHRS7C	− 3.118	6.95E − 13	LINC02616	− 2.736	1.38E − 08
	LINC00460	3.325	1.04E − 11	SYT10	− 2.551	1.65E − 08
	KRT24	− 3.688	1.18E − 11	SPATA12	1.586	1.87E − 08
	VSTM2A-OT1	− 2.434	2.16E − 10	TM4SF19	2.436	2.40E − 08
	CST2	3.382	2.61E − 10	MPP4	2.438	2.76E − 08
	HMX3	− 2.567	4.64E − 10	CA7	− 1.676	3.41E − 08
	NPY2R	− 3.139	8.97E − 10	LINC02023	− 2.826	3.67E − 08
	KHDRBS2	− 2.859	1.39E − 09	ZNF492	− 2.531	3.83E − 08
SGs-LE	LINC02691	− 1.925	1.01E − 05	BHLHA15	− 1.035	0.002
	MIR27B	− 1.731	1.67E − 05	ZPBP	1.596	0.002
	LY6K	− 1.820	5.84E − 05	HAPLN1	− 1.226	0.003
	IGF2-AS	2.134	6.39E − 05	CLPSL2	1.265	0.003
	SLC10A4	− 1.106	2.98E − 04	LSMEM2	− 1.144	0.003
	ADAMTS9-AS2	− 1.282	4.56E − 04	ACKR4	− 1.184	0.006
	H2AC13	− 1.175	5.24E − 04	LOC101927972	1.392	0.007
	ERICH4	1.660	8.61E − 04	LOC112267895	1.044	0.008
	CDC20B	1.241	9.52E − 04	TBX20	1.523	0.008
	ABCD2	− 1.359	0.001	LOC105373878	− 1.070	0.009

*SGs-LC* specific genes in Lynch syndrome and colorectal cancer, *SGs-LE* specific genes in Lynch syndrome and endometrial cancer

### The enriched GO terms and pathways

**Fig. 3:**
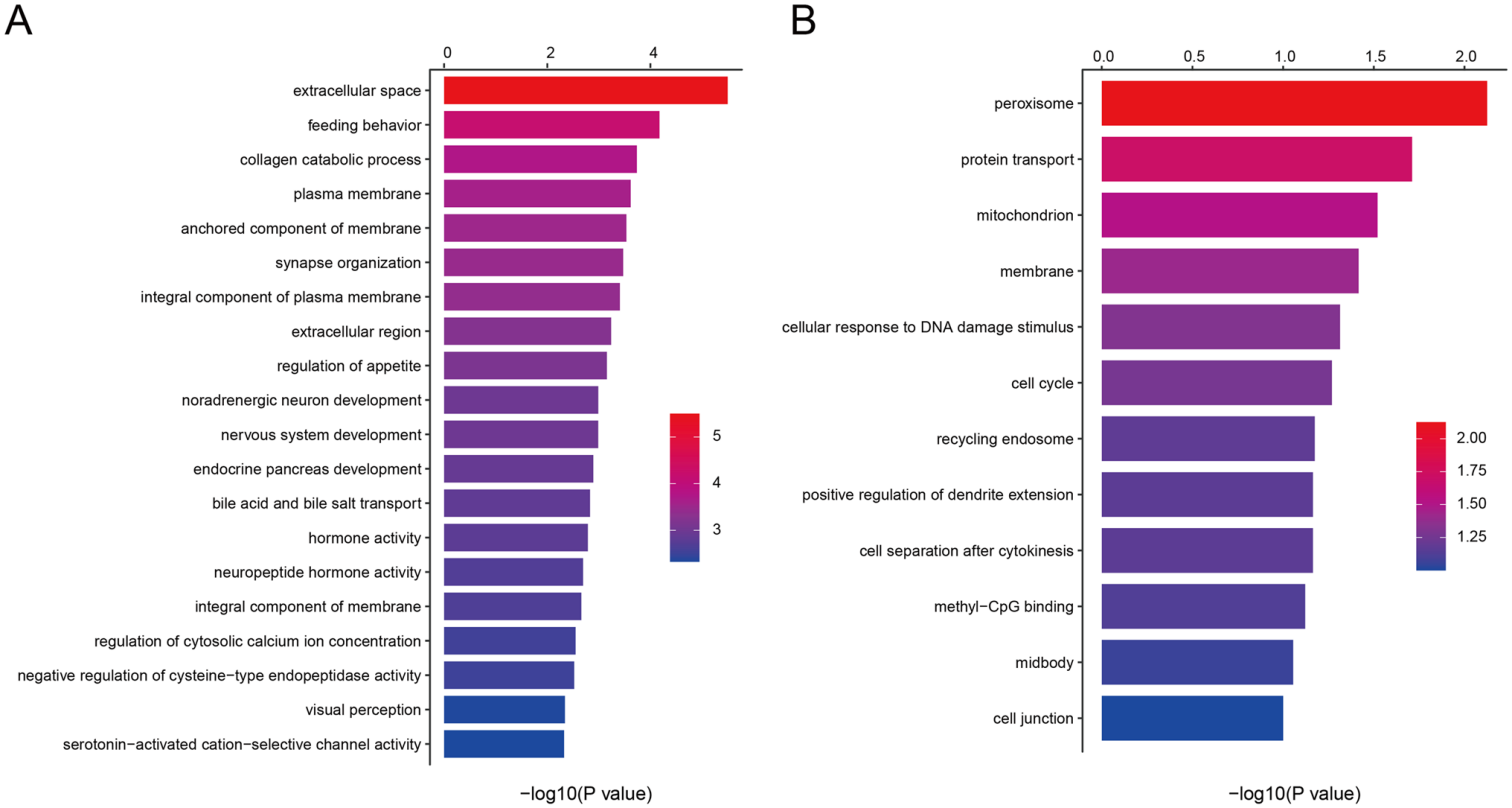
The top 20 enriched GO terms of SGs-LCs (**a**) and all GO terms enriched in SGs-LEs (**b**). *GO*, gene ontology; *SGs-LC*, specific genes in Lynch syndrome and colorectal cancer; *SGs-LEs*, specific genes in Lynch syndrome and endometrial cancer

After enrichment analysis, SGs-LCs were found to be enriched in 7 KEGG pathways and 106 GO terms, including 60 biological processes (BPs), 22 cellular components (CCs), and 24 molecular functions (MFs). The main enriched BPs were feeding behavior, collagen catabolic process, synapse organization and regulation of appetite. Enriched CCs were extracellular space, plasma membrane, anchoring component of membrane, and so on. Enriched MFs were hormone activity, neuropeptide hormone activity, serotonin-activated cation-selective channel activity, and bile acid transmembrane transporter activity. Figure 3a shows the top 20 GO terms according to *p* value. The KEGG pathways of SGs-LCs were significantly enriched in serotonergic synapse, maturity onset diabetes of the young, PPAR signaling pathway, and arrhythmogenic right ventricular cardiomyopathy (ARVC) (Table 2).

**Table 2:** SGs-LC- and SGs-LE-enriched KEGG pathways

Group	Pathway	Count	*p* value
SGs-LC	hsa04726:Serotonergic synapse	9	8.32E−04
	hsa04950:Maturity onset diabetes of the young	4	0.011
	hsa03320:PPAR signaling pathway	5	0.032
	hsa05412:Arrhythmogenic right ventricular cardiomyopathy (ARVC)	5	0.032
	hsa04080:Neuroactive ligand-receptor interaction	10	0.062
	hsa04970:Salivary secretion	5	0.069
	hsa04974:Protein digestion and absorption	5	0.074
SGs-LE	hsa04146:Peroxisome	3	0.050

*SGs-LC* specific genes in Lynch syndrome and colorectal cancer, *SGs-LE* specific genes in Lynch syndrome and endometrial cancer

SGs-LEs were enriched in 12 GO terms, including 5 BPs, 6 CCs, and 1 MF. The GO terms are shown in Fig. 3b, among which peroxisome, mitochondrion, protein transport, cellular response to DNA damage stimulus, and membrane had *p* values less than 0.05. The only KEGG pathway identified as enriched among the SGs-LEs was the peroxisome pathway; however, the enrichment was not statistically significant (*p* = 0.050, Table 2). There was a great difference in enriched GO terms and pathways between SGs-LCs and SGs-LEs, and SGs-LCs were enriched in more pathways, indicating that LS might likely develop into CRC through more pathways, consistent with our previous speculation.

### Protein–protein interaction networks

**Fig. 4:**
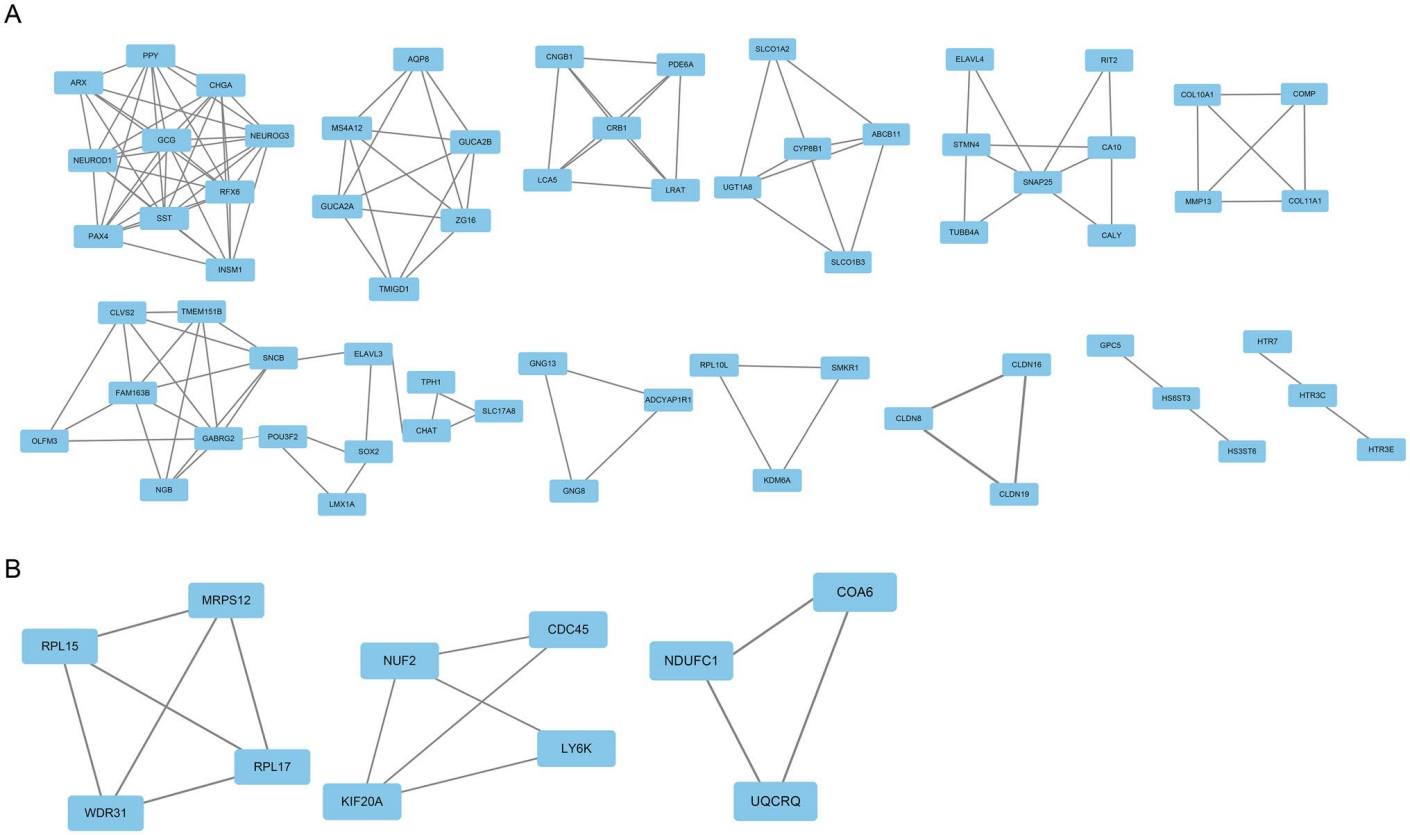
PPI network of SGs-LCs and SGs-LEs. SGs-LCs and SGs-LEs formed 11 clusters and 2 clusters, containing 81 and 8 genes, respectively. *PPI* protein–protein interaction; *SGs-LCs*, specific genes in Lynch syndrome and colorectal cancer; SGs-LEs, specific genes in Lynch syndrome and endometrial cancer

After STRING analysis, 663 interaction pairs between 280 proteins were obtained in SGs-LCs. These pairs formed 12 clusters and contained 66 genes (Fig. 4a). *SNAP25, SST*,* GCG,* and GABRG2 were involved in most pairs. Table 3A shows the top 20 genes with the highest degrees in the network. A total of 24 interactions were obtained from SGs-LEs. In the whole network, there were 3 clusters containing 11 genes with degree ≥ 2 (Fig. 4b), of which *KIF20A* and *NUF2* had the highest degrees (Table 3B). These genes could be potential key genes for the development of LS into CRC or EC.

**Table 3:** The nodes of the PPI network with high degree in lynch syndrome–colorectal cancer and lynch syndrome–endometrial cancer-specific genes

Gene	Degree	Gene	Degree
A. Top 20 nodes in Lynch syndrome–colorectal cancer-specific genes			
SNAP25	33	GAD1	17
SST	27	SOX2	17
GCG	26	ASCL1	16
GABRG2	26	ELAVL3	16
NEUROD1	21	NEUROG3	15
CHGA	21	ELAVL4	15
SYT4	21	PYY	14
CA10	20	OLFM3	13
NPY	18	TPH1	13
SNCB	18	CHAT	12
B. 8 nodes with degree ≥ 2 in Lynch syndrome–endometrial cancer-specific genes			
NUF2	3	RPL17	3
KIF20A	3	CDC45	2
COA6	3	NDUFC1	2
MRPS12	3	UQCRQ	2
RPL15	3	LY6K	2
WDR31	3		

*PPI* protein–protein interaction

### Correlation between gene expression level and survival

**Fig. 5:**
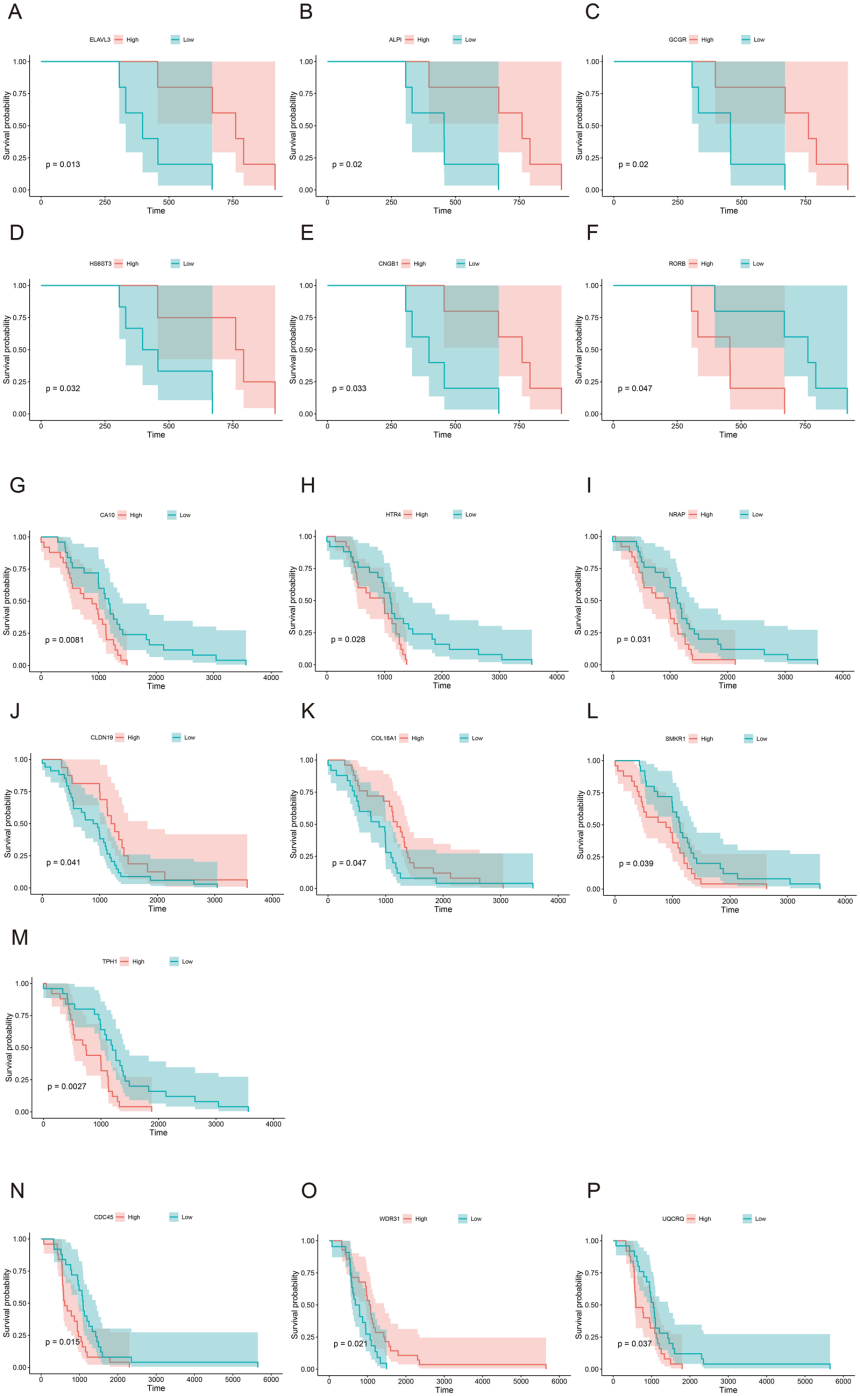
Genes with significant differences in survival rates between SGs-LCs and SGs-LEs at high or low expression levels. Among the SGs-LCs, significantly different survival rates of Lynch syndrome patients were found for high vs. low expression levels of *ELAVL3* (**a**), *ALPI* (**b**), *GCGR* (**c**), *HS6ST3* (**d**), *CNGB1* (**e**), and *RORB* (**f**); the survival rates of colorectal cancer patients with high vs. low expression of *CA10* (**g**), *HTR4* (**h**), *NRAP* (**i**), *CLDN19* (**j**) and *COL18A1* (**k**) were significantly different. In EC, high and low expression levels of *CDC45* (**l**) and *WDR31* (**m**) were associated with significantly different survival rates in endometrial cancer patients. SGs-LCs, specific genes in Lynch syndrome and colorectal cancer; SGs-LEs, specific genes in Lynch syndrome and endometrial cancer

In SGs-LCs, the genes with a significant difference in survival rate among LS patients with high expression levels and low expression levels were *ELAVL3* (*p* = 0.013), *ALPI* (*p* = 0.020), *GCGR* (*p* = 0.020), *HS6ST3* (*p* = 0.032), *CNGB1* (*p* = 0.033), and *RORB* (*p* = 0.047) (Fig. 5a–f). The expression levels of *CA10* (*p* = 0.008), *HTR4* (*p* = 0.028), *NRAP* (*p* = 0.031), *CLDN19* (*p* = 0.041), *COL18A1* (*p* = 0.047), SMKR1 (*p* = 0.039), and TPH (*p* = 0.0027) were significantly correlated with the survival rates of CRC patients (Fig. 5g–m). In SGs-LEs, there was no significant difference in survival rates between LS patients with high and low expression of any SGs-LE gene. In EC, the genes with significantly different survival rates between patients with high and low expression levels were *CDC45* (*p* = 0.015), *WDR31* (*p* = 0.024), and *UQCRQ* (*p* = 0.037) (Fig. 5n–p). These genes might be key genes for the prognosis of patients with LS, CRC, and EC.

### Verification of the specific genes

MSI is caused by a defect in one of the MMR genes and is strongly related to tumorigenesis. The MMR genes (*MLH1*, *MSH2*, *MSH6*, and *PMS2*) are pathogenic genes in LS. Therefore, we first analyzed the mutations in SGs-LCs and SGs-LEs in CRC and EC patients with MSI-H and MSS. *COL11A1* is associated with malignancy in colorectal cancer. In this study, *COL11A1* was identified in SGs-LC and exhibited one missense mutation, three frameshift mutations, and one intron mutation in MSS CRC patients. In addition, the mutation profile of *COL11A1* was very similar to that of *MSH6*, as both contained intron, missense, and frameshift mutations; therefore, *COL11A1* may be the key gene to distinguish MSI-H and MSS CRC patients.

**Fig. 6:**
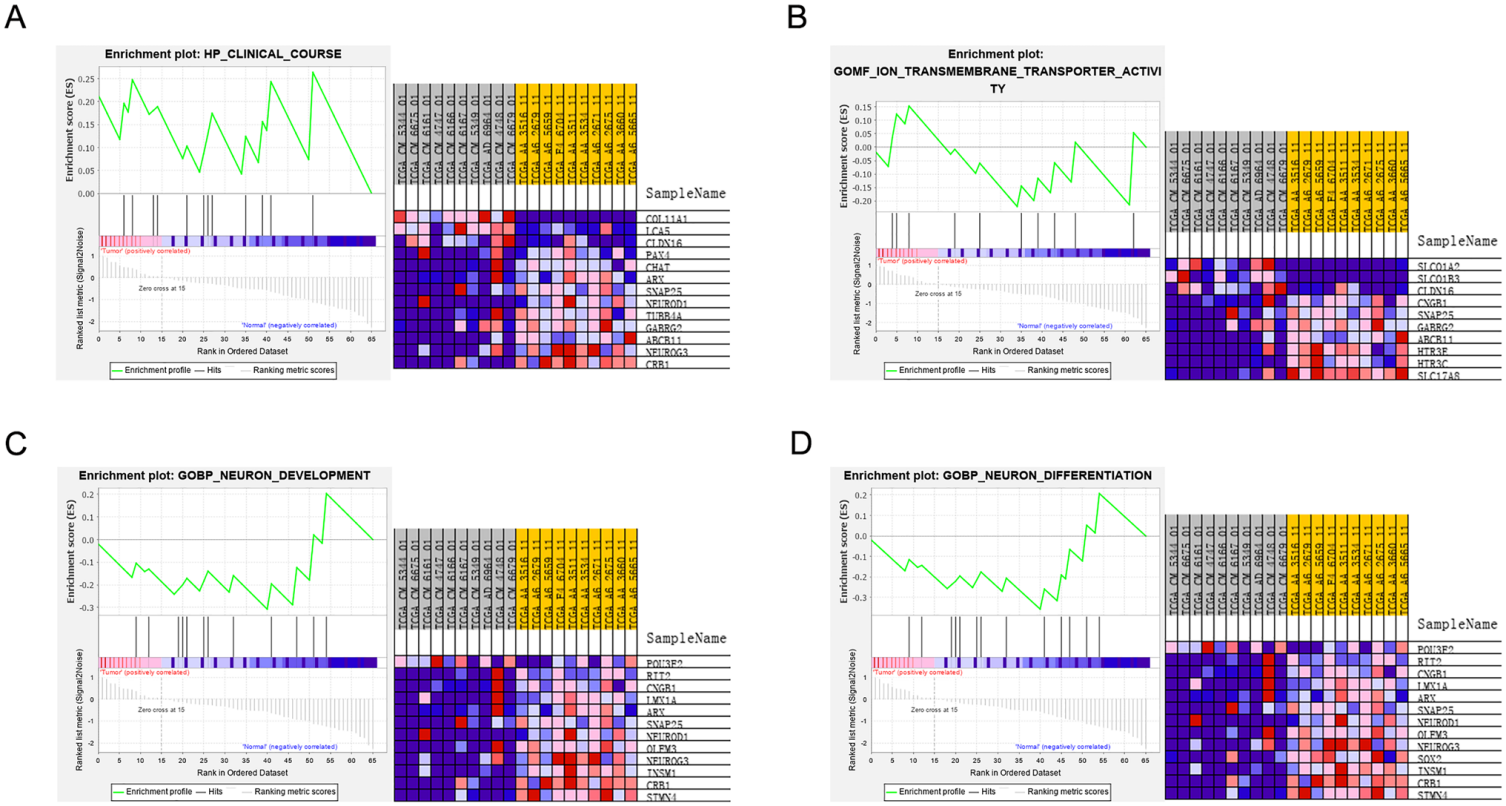
GSEA was carried out to identify upregulated or downregulated GO gene sets. SGs-LCs were differentially enriched in the Lynch syndrome-related GO terms of neurogenesis (**a**) and regulation of cell differentiation (**b**) and the GO terms cell_cell signaling **c**, ion transport **d**, neuron projection **e** and regulation of cell differentiation (**f**) related to colorectal cancer. Red shading in the heatmap represents upregulation, and blue represents downregulation. *GSEA* gene set enrichment analysis, *GO* gene ontology, *SGs-LCs* specific genes in Lynch syndrome and colorectal cancer

Finally, we analyzed the expression of SGs-LC and SGs-LG in normal and tumor samples in LS-, CRC-, and EC-related pathways through GSEA of GO gene sets. SGs-LE was not significantly different between normal and tumor samples. The differentially expressed genes in SGs-LC were enriched in 4 GO terms:HP_clinical_course, GOMF_ion_transmembrane_transporter_activity, GOBP_neurogenesis, GOBP_neuron_differentiatiation (Fig. 6a–d).

## Discussion

LS is an autosomal dominant disease. Carriers of pathogenic MMR mutations have a lifetime risk of approximately 30–70% for CRC, and the risk of EC in female carriers is approximately 30–60% [11]. The risk of other malignant tumors, such as gastric cancer, pancreatic cancer, and urinary system tumors, is also significantly higher in MMR mutation carriers than that in the general population [12, 13]. Patients with LS are at higher risk of gastrointestinal, gynecological, skull, and skin cancers, the most common of which are CRC and EC.

Although some progress has been made in the mutation of MMR genes in CRC and EC, the molecular mechanism and comparison of LS to CRC or EC has not been reported [14]. In this study, we analyzed the differential expression, differential methylation, and mutation of genes in LS, CRC, and EC from the TCGA database. There were significantly more specific DEGs in LS and CRC than in LS and EC. However, the specific DMGs showed the opposite result, indicating that the development of LS into CRC may occur through differential gene expression, while the development into EC may occur through gene methylation (Fig. 2a, b). Specific mutations were less frequent in both LS-CRC and LS-EC, and the proportion of mutation types was significantly different in LS, CRC, and EC patients; it was indicated that missense mutation may have some influence on the development of LS into cancers but was not the main mechanism (Fig. 2c, d).

By analyzing the correlation of DEGs, DMGs, and mutations with the LS pathogenic genes *MLH1*, *MSH2*, *MSH6*, *PMS2*, and *EPCAM*, we finally obtained 493 specific genes for LS and CRC (SGs-LC) and 99 specific genes for LS and EC (SGs-LE). The results of GO enrichment analysis showed that SGs-LCs and SGs-LEs were enriched in different GO terms and KEGG pathways, and the numbers were quite different. Although the enriched pathways of SGs-LCs and SGs-LEs were different, they both included pathways related to peroxisomes, which revealed that there might be correlations between the mechanisms of LS developing into CRC and EC as well as certain differences. These results provide insights into the molecular mechanism by which LS progresses to CRC or EC.

Through the construction of the PPI network, we obtained 4 (*SNAP25, SST, GSG, GSBRG2*) and 2 (*NUF2,KIF20A*) genes with high node degrees in SGs-LC and SGs-LE, respectively. *SST* (somatostatin) was isolated and purified from the sheep hypothalamus in 1973. Subsequent studies have confirmed that somatostatin not only exists in the hypothalamus but is also widely distributed in the brain, pancreas, and intestinal nerve cells. *SST* is a regulatory peptide that functions in exocrine, endocrine, paracrine, and autocrine processes. It has a wide range of biological activities and plays an important role in regulating the physiological functions and pathogenesis of some diseases. *SST* can not only inhibit the hormone production of endocrine cells but also inhibit the mitosis of cells. Most experimental animals and human cell lines have shown that *SST* can inhibit the growth of tumors such as pancreatic cancer, CRC, and liver cancer [15, 16]. *NPY* (neuropeptide Y) is a sympathetic neurotransmitter belonging to the pancreatic polypeptide family, and a single-nucleotide polymorphism (SNP) associated with this gene is related to hypertension, obesity, diabetes, and cardiovascular disease [17]. *KIF20A* (Kinesin 20A), a member of the kinesin superfamily, is mainly involved in mitosis [18]. Recently, abnormal *KIF20A* expression has been found in breast cancer, cervical squamous cell carcinoma, and hepatocellular carcinoma, suggesting that this gene is closely related to the formation and development of tumors [19–22]. *NUF2*, a cell division-related gene, plays a role in stabilizing centromeres and ensuring accurate separation of chromosomes during mitosis [23]. Studies have found that *NUF2* is highly expressed in a variety of malignant tumors, including lung cancer, liver cancer, CRC, and gastric cancer, and plays an important role in tumor formation and development [24–26]. These genes may be the key genes for the development of LS into CRC and EC.

Furthermore, we analyzed the effect of SGs-LCs and SGs-LEs on patient survival. After screening, we found that the expression levels of different genes in LS, CRC and EC patients were quite different, and the genes affecting the survival of patients were also different. The expression levels of *ALPI*, *CNGB1*, *ELAVL3*, *GCGR*, *HS6ST3*, and *RORB* in SGs-LC were significantly correlated with the survival rate of patients with LS and *CA10*, *CLDN19*, *COL18A1*, *HTR4*, SMKR1,TPH1, and *NRAP* with CRC. In SGs-LE, there was a significant difference in the survival rate between endometrial carcinoma patients with high and low expression levels of *CDC45, UQCRQ*, and *WDR31*. Previous studies have reported some of these genes in cancer. Takashi et al. [27] detected *GCGR* expression in CRC tissues, and downstream signals of *GCGR*, including AMPK and MAPK pathways, play vital roles in the proliferation of CRC in vitro and in vivo. *HTR4* performs an important function in human prostate cancer through 5-HT secreted by cancerous tumors and mast cells surrounding the tumors. In addition, in prostatic cancer, estrogen may regulate the effect of *HTR4* through estrogen receptor β (ERβ) to affect the development of cancer [28]. *COL18A1* encodes XVIII collagen, which is a widely expressed nonfibrillar collagen and an important component of the extracellular matrix (ECM), which was consistent with our enrichment analysis finding that *COL18A1* was enriched in the GO term of extracellular space. A study found that the risk of sporadic breast cancer was significantly increased in patients with the *COL18A1* D104N polymorphism [29]. In addition, the upregulated expression of *COL18A1* was found in bladder cancer patients with tumor stages T1 and T2, which may be involved in the progression of bladder cancer by affecting extracellular matrix-receptor interactions and adhesion sites [30]. *CDC45* is a component of the cell division cycle 45 (*CDC45*) minichromosome maintenance protein complex (MCM) and has been reported to be upregulated and identified as a hub gene in non-small-cell lung cancer [31]. *CDC45* might also promote papillary thyroid cancer progression and nasopharyngeal carcinoma [32, 33]. These genes may be potential prognostic markers for LS, CRC and EC.

Finally, we compared the mutations of these genes in CRC and EC patients with MSI-H and MSS and their correlation with dislocation mutations in five pathogenic genes of LS. Microsatellites are short tandem repeats that are present throughout the human genome. The length of microsatellites changes in tumor cells with respect to that in normal cells due to the insertion or deletion of repeat units; this process is called MSI. Numerous studies have shown that MSI is caused by defects in MMR genes and is closely related to tumorigenesis. MSI has been clinically used as an important molecular marker for the prognosis and adjuvant treatment of CRC and other solid tumors and has been used to assist in screening for LS. In this study, *COL11A1* exhibited 1 missense, three frameshift, and one intron mutation in MSS CRC. The correlation analysis results showed that *COL11A1* mutation spectrum was consistent with that of *MSH6* mutations and had a strong correlation with *MSH6* mutation. The *COL11A1* gene is located in the chromosome 1p21 region, contains 68 exons, and is mainly expressed in articular cartilage. Recent studies have found that *COL11A1* plays an important role in the occurrence and development of tumors, and its expression is upregulated in head and neck squamous cell carcinoma and in ovarian, gastric, and pancreatic cancer [34–37]. Our results illustrated that *COL11A1* may be a marker for the differentiation of MSI-H and MSS CRC patients and for the development of LS into MSS CRC. In addition, all of the four validation GO terms enriched in SGs-LCs in GSEA database are related to CRC. Our results revealed few verified genes and GO terms. The possible reasons for this are as follows: first, the number of patients we collected was small, and the gene mutation detection was not comprehensive; second, most of the data in TCGA are from Caucasian populations, whereas the patients we analyzed were all from Asian populations. The difference in race may also be one of the reasons for the difference in mutation type and frequency. More experimental research is needed to confirm the current findings.

In brief, we obtained genes specific to LS-CRC and LS-EC, which may be key genes involved in the progression of LS to CRC and EC. At the same time, our study reveals that the molecular mechanisms of LS development to CRC and EC are different. Since cancer is the result of multiple highly complex molecular mechanisms, a single pathway is not sufficient to explain cancer pathogenesis [38]. However, our findings provide novel insights into the mechanisms by which LS develops into CRC or EC and their differences.

## Supplementary Information

Below is the link to the electronic supplementary material.

Supplementary file1 (DOC 135 kb)

## Data Availability

The datasets used and/or analyzed during the current study are available from the corresponding author on reasonable request.
