# Supplementary material for: Molecular mechanisms and differences in lynch syndrome developing into colorectal cancer and endometrial cancer based on gene expression, methylation, and mutation analysis
Source: Cancer Causes Control. 2022 Feb 11;33(4):489–501. doi: 10.1007/s10552-021-01543-w (PMC8904372; doi:10.1007/s10552-021-01543-w)
Supplement: Supplementary file 1 — Supplementary file1 (DOC 135 kb) [file 10552_2021_1543_MOESM1_ESM.doc]

Supplementary Table 1A The 493 specific genes for Lynch syndrome and colorectal cancer

| BCL9L | SALL4 | KCNB2 | MIR8071-2 | ATCAY | RPL10L | NUPR2 |
| --- | --- | --- | --- | --- | --- | --- |
| BNC2 | LY6G6F | ANKS1B | REP15 | MOBP | ECRG4 | RFX6 |
| CACNA1G | CSTL1 | HTR3A | ALPI | LRRTM1 | MYOC | LOC285804 |
| COL18A1 | FBXW12 | CELF4 | UBXN10-AS1 | KRT9 | CNTN5 | CHST9 |
| EIF2AK1 | LINC00922 | SNAP25 | LINC00877 | CASC16 | LOC105372441 | GPR15 |
| FOLH1 | RPSAP52 | LINC01354 | GFRA2 | LINC00582 | DUSP21 | ZDHHC22 |
| HIF3A | CST5 | SCN11A | PRAMENP | LINC01954 | FOXD3 | GNG13 |
| KCNG1 | LMO7-AS1 | B3GALT1 | SYT4 | LRAT | IL1RAPL1 | RALYL |
| KDM6A | TTLL9 | B3GNT6 | TMEM155 | ADCYAP1R1 | ARPP21 | KCTD4 |
| MEGF6 | MMP7 | PCDH10 | MAL | SLCO4C1 | SVOPL | ST8SIA3 |
| MEX3B | IFITM5 | GBA3 | DNER | PWRN1 | GPC5 | INSM1 |
| NEXMIF | SNORD12B | SPIB | KCNJ16 | PWRN3 | CUX2 | PPY |
| PCDHB7 | SMKR1 | KCNK2 | SLC7A3 | LVRN | CHL1-AS2 | LOC102724153 |
| PFAS | RAD51AP2 | TMEM100 | FAM218A | WI2-2373I1.2 | NELL1 | LINC02490 |
| PTPN11 | LOC101928222 | MT1M | FAM163B | LRP1B | FYB2 | IQCM |
| SIPA1L3 | CACNG8 | NAP1L2 | CNR2 | ZMAT4 | MIR7-3HG | LINC01645 |
| TCF7L2 | IGFL2-AS1 | AQP12A | LINC00940 | SCGB2A1 | MRGPRX2 | PHOX2B |
| URGCP | SMCO2 | SI | CYP8B1 | KLHL34 | TEX11 | LMX1A |
| ZNF16 | LOC107984125 | ABCA8 | LINC02158 | KBTBD12 | LOC105379030 | LINC00682 |
| CST1 | RP1 | LINC00964 | CACNG7 | LINC02293 | MT1JP | VSTM2A-OT1 |
| CST2 | SPATA12 | LINC02292 | NKAIN3-IT1 | FCER2 | NRSN1 | SLC30A10 |
| LINC00460 | LINC02254 | ELAVL4 | UGT1A9 | TMEM132D | NPY | CHAT |
| SFTA2 | GSC | FAM181B | CCDC178 | CA10 | GABRG2 | SLC17A8 |
| KLK6 | COMP | CNTN6 | MYPN | XXYLT1-AS2 | PAX4 | SLC13A1 |
| FIRRE | FABP6 | CCNA1 | BEST2 | CLDN19 | PHOX2A | PYY |
| FEZF1-AS1 | FLJ16779 | KRBOX1 | HTR7 | LINC01634 | IL5RA | ZNF492 |
| EPYC | CXCL17 | LIPC | TUBB4A | SULT4A1 | NRAP | NGB |
| MMP13 | AGRP | SVOP | IGSF11 | ARX | SLC4A10 | SYT10 |
| TCF24 | AKR1C4 | LINC01014 | FGFBP2 | AMER3 | UNC5D | HMX3 |
| LEMD1 | LOC730668 | GUCA2A | LRRC18 | CNTFR | PKHD1L1 | HS3ST6 |
| SLCO1B3 | LOC100128059 | SCG3 | DPYSL5 | KLRF1 | SGCG | TMIGD1 |
| F2 | PTRH1 | RIT2 | TAS1R1 | UGT1A8 | SLC7A14 | LINC00974 |
| MPP4 | STRA6 | BEST4 | RORB | MS4A12 | GS1-204I12.4 | CDH10 |
| TM4SF19 | DCXR-DT | ZG16 | TMEM151B | CIDEA | TTR | LINC02000 |
| PCAT2 | LEF1-AS1 | TNFRSF13B | FER1L6 | FCRL4 | KLK13 | GUCA2B |
| IGFL4 | PGC | RPH3A | CPNE4 | SPX | LRRC3B | OLFM3 |
| DMRT3 | POU3F2 | PRIMA1 | TPH1 | SPINK2 | LOC105378318 | PCAT18 |
| LOC101929705 | DIAPH2-AS1 | CTNNA2 | MAP1LC3C | KRT27 | LINC00461 | HTR3C |
| FEZF1 | TRIM72 | LRRTM4 | GNG8 | SLC6A15 | SST | MMP27 |
| PRDM12 | SNORD67 | LINC02408 | CHST5 | LOC729558 | TCEAL5 | LINC02616 |
| COL10A1 | TMEM40 | LOC100240735 | COPDA1 | AQP8 | XKR4 | LINC01687 |
| SLCO1A2 | C20orf144 | PTCHD1 | MLIP | LOC101927495 | P2RX2 | LINC02023 |
| LINC00618 | SNORA22B | LINC01082 | CTNND2 | CALY | CPB1 | KHDRBS2 |
| CRAT37 | ST7-OT4 | BMP3 | SEMA3E | TNFRSF17 | GSG1L | KRTAP13-2 |
| DSCR9 | CRNDE | LOC101928228 | STXBP5L | FUT9 | TBPL2 | DAO |
| FSIP2-AS2 | LOC101927480 | CHODL | LINC01505 | SCGN | LOC284191 | HTR3E |
| TAS2R38 | DSG1-AS1 | CCL13 | HNRNPA1P33 | CA7 | LHFPL4 | ABCB11 |
| SNORD15B | HMGA2-AS1 | KCNIP1 | ABCC13 | MEP1B | C11orf86 | LINC00507 |
| LINC02099 | COL11A1 | SIGLEC11 | UTS2B | OR2W3 | PROKR1 | VSTM2A |
| LINC01101 | AKAP4 | GALNT13 | ACADL | SLC16A12 | NEUROG3 | NEUROD1 |
| LBP | GAD1 | HS6ST3 | C9orf135 | ROPN1 | RXRG | AADACL2 |
| HABP2 | KRT80 | CD177 | BFSP2 | TRPC7 | LOC102724957 | DHRS7C |
| LINC01615 | SPRY4-AS1 | TMEFF2 | CBLN2 | CLVS2 | SLC27A6 | OTOP3 |
| MIR4713HG | CLDN16 | VXN | TAT | LINC01013 | SFRP5 | NPY2R |
| NPSR1-AS1 | FOXQ1 | CXCR5 | LEXM | IGLL1 | SOX2 | GCG |
| C2-AS1 | FARSA-AS1 | PDE6A | DIRAS2 | POU3F4 | RIMS4 | INSL5 |
| LOC100996351 | FGF17 | GPR88 | ELAVL3 | FOXD3-AS1 | SNCB | CLDN8 |
| WNT2 | C9orf50 | ENHO | SHOX | BEND4 | OSTN-AS1 | KRT24 |
| ESM1 | TAL2 | PIRT | HOXD1 | NCR2 | ERICH3 | LOC100130264 |
| LOC101927858 | DHRS2 | FCRLA | LOC100130449 | ASCL1 | CRYBA2 | SNRPD2 |
| LINC02072 | MIR34AHG | SLC17A1 | NSG2 | DPP10 | STMN4 | TRAPPC3 |
| LINC02073 | MIR31HG | PCDHA1 | LOC100505851 | SH2D7 | LINC00402 | ARID4A |
| EXTL3-AS1 | PTGES2-AS1 | ECI2-DT | HTR4 | LINC01781 | CA1 | LCA5 |
| IRX5 | PKP4-AS1 | TARID | GP2 | IL2 | P2RY4 | ZNF140 |
| LPO | TNNT2 | CWH43 | CHGA | ERBB4 | MUSK | MIR761 |
| SPAG17 | EPHX4 | CNTN3 | GCGR | PLAC9P1 | CLCA2 | RAB6C |
| INHBA-AS1 | CLEC5A | NTNG1 | DPEP3 | CRB1 | PIK3C2G | LOC341056 |
| STPG4 | IFNE | CNGB1 | COL19A1 | ASTN1 | LINC02588 | GNPDA2 |
| LINC02345 | SNORA69 | CAV3 | CDKN2B-AS1 | SEMA6A-AS2 | LOC100506289 | ZFC3H1 |
| DIRC1 | CCDC78 | KCNA3 | SMIM28 | RASGEF1C | ITPRID1 | MIR943 |
| MIR107 | PGBD1 | CYP2J2 |  |  |  |  |

Supplementary Table 1B The 99 specific genes for Lynch syndrome and endometrial cancer

| CNTNAP4 | CRYBG3 | DMBT1 | DOCK2 | ERCC6L2 | GPR85 | PSME4 |
| --- | --- | --- | --- | --- | --- | --- |
| RIMS2 | SEMA6B | SMURF1 | WDR66 | IGF2-AS | ERICH4 | ZPBP |
| TBX20 | LOC101927972 | TUBB8 | CLPSL2 | CDC20B | LOC93429 | LOC112267895 |
| BHLHA15 | LOC105373878 | SLC10A4 | LSMEM2 | H2AC13 | ACKR4 | HAPLN1 |
| LOC100128325 | ADAMTS9-AS2 | MIRLET7D | ABCD2 | MIR27B | LY6K | LINC02691 |
| SNORD115-8 | OR4S2 | GPAA1 | C3orf10 | OSTM1 | MRPS12 | MITD1 |
| LIN37 | PCSK7 | CTSC | SCO2 | PEX11A | C2orf44 | UQCRQ |
| ERP29 | RPL15 | ACP2 | NDUFC1 | FUNDC2 | PDE7A | CCNK |
| NAA35 | GON4L | ARMCX6 | PRRT2 | TMTC3 | MED30 | HSDL2 |
| CDC45L | RBM7 | MIR135A1 | U58 | KIAA0090 | ARFGEF2 | TMEM39B |
| PDHA1 | CYB5D2 | RINT1 | RBM12 | ZNF841 | DNAJA3 | SNORD24 |
| SNORD50A | TCEAL1 | C1orf31 | NUF2 | POP5 | ERH | IDH1 |
| UNC50 | MIR182 | KIF20A | SLC35A4 | ZBTB33 | ATG9A | PDCD2L |
| XIAP | GTF3C5 | PTPDC1 | NUP50 | ZDHHC21 | C11orf51 | WDR31 |
| APTX |  |  |  |  |  |  |
